# Acid Peptic Disease among Patients with Acute Abdomen Visiting the Department of Emergency Medicine in a Tertiary Care Centre

**DOI:** 10.31729/jnma.8228

**Published:** 2023-08-31

**Authors:** Rashmi Thapa, Mohir Pokharel, Saroj Paudel, Tunam Khadka, Priyanka Sapkota, Rohit Rana, Moneec Pokharel, Dinesh Chhetri

**Affiliations:** 1 Department of Emergency Medicine, Kathmandu Medical College and Teaching Hospital, Sinamangal, Kathmandu, Nepal; 2 Kathmandu Medical College and Teaching Hospital, Sinamangal, Kathmandu, Nepal; 3 Universal College of Medical Sciences, Bhairahawa, Rupandehi, Nepal; 4 Birat Medical College and Teaching Hospital, Biratnagar, Morang, Nepal; 5 Everest Hospital, New Baneshwor, Kathmandu, Nepal

**Keywords:** *acute abdomen*, *gastroenteritis*, *Helicobacter pylori*, *peptic ulcer*

## Abstract

**Introduction:**

Acid peptic disease is caused by excessive acid secretion or weakened mucosal defense. Symptoms include epigastric pain, bloating, and nausea. Factors like gastric acid, *Helicobacter pylori* infection, alcohol consumption, smoking, and stress contribute to peptic ulcers. Imbalances between offensive and defensive factors can lead to ulcers. Acid-related disorders impact the quality of life and mortality. Accurate diagnosis and prompt treatment are vital. This study aimed to find out the prevalence of acid peptic disease among patients with acute abdomen in the Department of Emergency Medicine in a tertiary care centre.

**Methods:**

A descriptive cross-sectional study was conducted from 2 April 2022 and 2 April 2023 among the patients presented in the Department of Emergency Medicine in a tertiary care centre. Ethical approval was obtained from the Institutional Review Committee. All patients presenting with acute abdominal pain in the Emergency Department were included in the study. Patients not giving consent were excluded from the study. Convenience sampling method was used. The point estimate was calculated at a 95% Confidence Interval.

**Results:**

Out of the 400 patients with acute abdomen, the prevalence of acid peptic disease was found to be 87 (21.75%) (17.71-25.79, 95% Confidence Interval).

**Conclusions:**

The prevalence of acid peptic disease among patients with acute abdomen was found to be lower than in other studies performed in similar settings.

## INTRODUCTION

Acid peptic disease is a clinical condition due to overlapping pathogenic mechanisms leading to either excessive acid secretion or diminished mucosal defense characterized by epigastric pain associated with dyspepsia, bloating, abdominal fullness, nausea, or early satiety.^1,2^ It is one of the commonest causes of acute abdomen. Acute abdomen is a frequent presentation in emergency departments worldwide, accounting for about 10% of all visits.^3^ Imbalances between offensive (acid, Helicobacter pylori, smoking, stress) and defensive (mucosa, blood fluid, prostaglandins, antioxidants) factors can also lead to ulcers.^4^

Acid-related disorders influence the quality of life and productivity of afflicted patients and are common and important causes of morbidity and mortality.^1^ So, accurate diagnosis and prompt medical management are crucial.

This study aimed to find out the prevalence of acid peptic disease among patients with acute abdomen in the Department of Emergency Medicine in a tertiary care centre.

## METHODS

A descriptive cross-sectional study was conducted on patients presenting to the Department of Emergency Medicine at Kathmandu Medical College and Teaching Hospital, Sinamangal, Kathmandu, Nepal from 2 April 2022 to 2 April 2023. Ethical approval was taken from the Institutional Review Committee (Reference number: 100920186). All patients presenting with acute abdominal pain in the Emergency Department were included in the study after giving consent. Patients not giving consent were excluded from the study. Convenience sampling method was used. The sample size was calculated by using the following formula:


$$\begin{array}{ll} \text{n} & ={\text{Z}}^{2}\times \frac{\text{p}\times \text{q}}{{\text{e}}^{\text{2}}}\\ & =1.96^{2}\times \frac{0.50\times 0.50}{0.05^{2}}\\ & =385 \end{array}$$

Where,

n = minimum required sample size
Z = 1.96 at 95% Confidence Interval (CI)
p = prevalence taken as 50% for maximum sample size calculation
q = 1-p
e = margin of error, 5%

The minimum required sample size was 385. However, 400 samples were included in the study. The diagnosis of acid peptic disease was based on the clinical symptoms in most of the patients. The most common presenting symptom of peptic ulcer disease is epigastric pain, which may be associated with dyspepsia, bloating, abdominal fullness, nausea, or early satiety. Most cases of peptic ulcer disease are related with *Helicobacter pylori* infection or the use of nonsteroidal anti-inflammatory drugs.^2^

Data were entered in Microsoft Excel 2016 and analyzed using IBM SPSS Statistics version 21.0. The point estimate was calculated at a 95% Confidence Interval.

## RESULTS

Among 400 patients with acute abdomen, the prevalence of acid peptic disease was found to be 87 (21.75%) (17.71-25.79, 95% CI). Among 87 patients, 23 (26.44%) had gastro-oesophageal reflux disease (GERD) and 64 (73.56%) had a peptic ulcer (Table 1).

**Table 1:** Acid peptic disease (n= 87).

Acid peptic disease	n (%)
GERD	23 (26.44)
Peptic ulcer	64 (73.56)
Zollinger-Ellison syndrome	-

**Figure 1:**
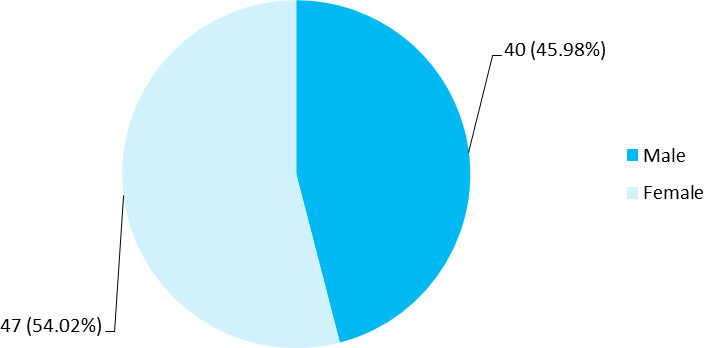
Gender-wise distribution (n = 87).

Zollinger-Ellison syndrome-Among 87 patients with acid peptic disease, 40 (45.98%) were male, and 47 (54.02%) were female (Figure 1).

The mean age of patients with acid peptic disease was 31.63±16.45 years. The most affected age group were 21-40 years, comprising 42 (48.28%) patients (Table 2).

**Table 2:** Age-wise distribution (n = 87).

Age (years)	n (%)
0-20	23 (26.44)
21-40	42 (48.28)
41-60	15 (17.24)
61-80	7 (8.05)

## DISCUSSION

In our study, the prevalence of acid peptic disease was found to be 87 (21.75%) out of 400 patients with acute abdomen which was lower than that of the study done in a similar setting. In a study among Nepalese patients, the prevalence was 48.60%.^5^

Acute abdomen is a medical emergency that can occur at any time in a lifetime of human beings and can put a substantial economic burden on a country where acid peptic disease occupies the majority portion.^6^ Relatively young and working age population was affected with a mean of 31.63±16.45 years, 21-40 years age group was the most affected age group, similar to the study done in other parts of the world.^7^ Our study showed the prevalence among elderly of more than 60 years affected to be only 7 (8.05%), which was relatively lower than other studies.^7^ Female predominance of 47 (54.02%) was seen as consistent with similar studies of 52.6% and 52%.^5,8^

Acid peptic disease, a condition causing significant morbidity and mortality worldwide, manifests in various ways. Interestingly, around two-thirds of patients diagnosed with peptic ulcer disease remain asymptomatic. However, for those who do experience symptoms, the most common presenting sign is epigastric pain, often accompanied by dyspepsia, bloating, abdominal fullness, nausea, or early satiety. The primary causes of peptic ulcer disease are Helicobacter pylori infection and the usage of nonsteroidal anti-inflammatory drugs (NSAIDs), or a combination of both. Additionally, psychological problems such as severe stress and depressed mood have been linked to the prevalence of acid peptic disease.^9,10^

Acid peptic disorders result from overlapping mechanisms causing excessive acid secretion or reduced mucosal defence. Medically histamine-2 receptor antagonists revolutionized treatment with their safety and efficacy, while proton-pump inhibitors offer stronger acid inhibition. Although PPIs reduce ulcer risk from NSAIDs, they cannot fully maintain an intragastric pH above 4.^1^ To prevent and heal acid peptic disease (APD), it's important to minimize the use of analgesic drugs and glucocorticoids, manage environmental and socioeconomic factors, maintain a balanced diet, engage in regular exercise, effectively cope with stress, abstain from smoking, limiting alcohol consumption, and ensuring sufficient sleep at night. By following these measures, one can significantly contribute to the prevention and recovery from APD.^11^

There is a high incidence of hospital admission due to acute abdominal pain, accounting for 23.50% in our study, which constitutes the majority of surgical causes; however, none of them got admitted because of acid peptic disease, whereas the overall incidence of acute abdomen leading to hospital admission ranges from 30% to 45%.^12^

Our study demonstrated that all patients with acid peptic disease were discharged from the ER after symptomatic management with proper medical counselling. However, our study had some limitations. It cannot be generalized as it was done at a tertiary level with a low sample size. Further studies needed to be done with a larger population sample to obtain more comprehensive data. The mentioned record might also be biased due to misdiagnosis and mishandling of records.

## CONCLUSIONS

The prevalence of acid peptic disease in acute abdomen was found to be lower than other studies done in similar settings. Hence, early diagnosis and medical management, and lifestyle modification are crucial to prevent complications and reduce the economic burden on the healthcare system.
